# Supplementary material for: The synthetic lethal interaction between CDS1 and CDS2 is a vulnerability in uveal melanoma and across multiple tumor types
Source: Nat Genet. 2025 Jul 4;57(7):1672–83. doi: 10.1038/s41588-025-02222-1 (PMC12283370; doi:10.1038/s41588-025-02222-1)
Supplement: Supplementary file 1 — Supplementary Figs. 1–15. [file 41588_2025_2222_MOESM1_ESM.pdf]

nature genetics

Supplementary information https://doi.org/10.1038/s41588-025-02222-1

# The synthetic lethal interaction between *CDS1* and *CDS2* is a vulnerability in uveal melanoma and across multiple tumor types

In the format provided by the authors and unedited

**SUPPLEMENTARY FIGURES**

# The synthetic lethal interaction between *CDS1* and *CDS2* is targetable across multiple tumor types

Pui Ying Chan[1†], Diana Alexander[1†], Ishan Mehta[1†], Larissa Satiko Alcantara Sekimoto Matsuyama[1†], Victoria Harle[1], Rebeca Olvera-León[1], Jun Sung Park[1], Fernanda G. Arriaga-González[1], Louise van der Weyden[1], Saamin Cheema[1], Vivek Iyer[1], Victoria Offord[1], David Barneda[2], Phillip Hawkins[2], Len Stephens[2], Zuza Kozik[3], Michael Woods[4], Kim Wong[1], Gabriel Balmus[1,4,5], Alessandro Vinceti[6], Nicola A. Thompson[1], Martin Del Castillo Velasco-Herrera[1], Lodewyk Wessels[7], Joris van de Haar[7], Emanuel Gonçalves[8], Sanju Sinha[9], Martha Estefania Vázquez-Cruz[10], Luisa Bisceglia[11], Francesco Raimondi[11], Jyoti Choudhary[3], Sumeet Patiyal[9], Anjan Venkatesh[12], Francesco Iorio[6], Colm J. Ryan[12], David J. Adams[1]*

**Affiliations:**

[1]Wellcome Sanger Institute, Hinxton, CB10 1SA, UK.
[2]Babraham Institute, Cambridge, CB22 3AT, UK.
[3]The Institute of Cancer Research. 123 Old Brompton Road. London SW7 3RP.
[4]UK Dementia Research Institute at the University of Cambridge and Department of Clinical Neurosciences, University of Cambridge, Cambridge, CB2 0AH, UK.
[5]Department of Molecular Neuroscience, Transylvanian Institute of Neuroscience, 400191, Cluj-Napoca, Romania
[6]Fondazione Human Technopole - Viale Rita Levi-Montalcini, 1 - Area MIND - 20157 Milano Italy.
[7]Netherlands Cancer Institute, Plesmanlaan 121, 1066CX Amsterdam.
[8]Instituto Superior Técnico (IST), Universidade de Lisboa, 1049-001, Lisbon, Portugal.
[9]Center for Cancer Research, National Cancer Institute, Bethesda, MD 20892, USA.
[10]Laboratorio Internacional de Investigación sobre el Genoma Humano, Universidad Nacional Autónoma de México, Campus Juriquilla, Querétaro, Qro, Mexico.
[11]Laboratorio di Biologia Bio@SNS. Scuola Normale Superiore. Piazza dei Cavalieri, 7, 56126 Pisa, Italy.
[12]University College Dublin, School of Computer Science, Belfield, Dublin 4, Ireland.

[†]These authors contributed equally.

Dr. David Adams*
Email: da1@sanger.ac.uk
Phone: +44 07826842781

**One Sentence Summary:** A synthetic lethal interaction exists between *CDS1* and *CDS2* that is targetable across multiple tumor types.

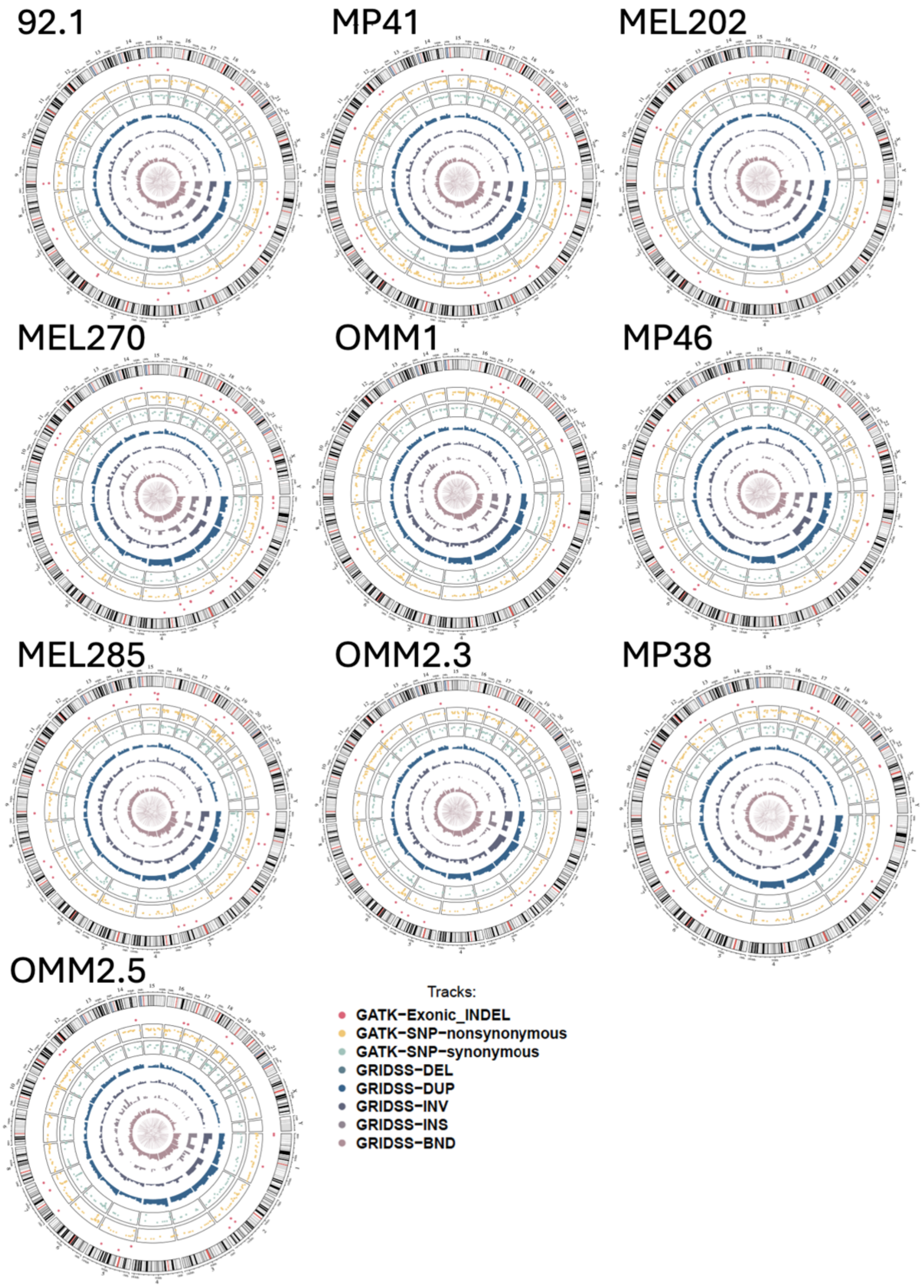


**Supplementary Fig. 1. Circos diagrams depicting the somatic mutational landscape of all 10 uveal melanoma cell line that underwent CRISPR screening and analysis.** The core displays the structural rearrangements. From the inside track out, the next four tracks display insertions followed by inversions, duplications and deletions in histogram form. The next two tracks out show single nucleotide variants (SNVs); synonymous SNVs are in green, non-synonymous SNVs in yellow. The next track shows exonic indels in red. The final outer track is the chromosome indicator; centromeres are in red, stalks (regions within the short arms of acrocentric chromosomes that are tightly constricted) are in blue. All variant calls are available for download from Github/Figshare.

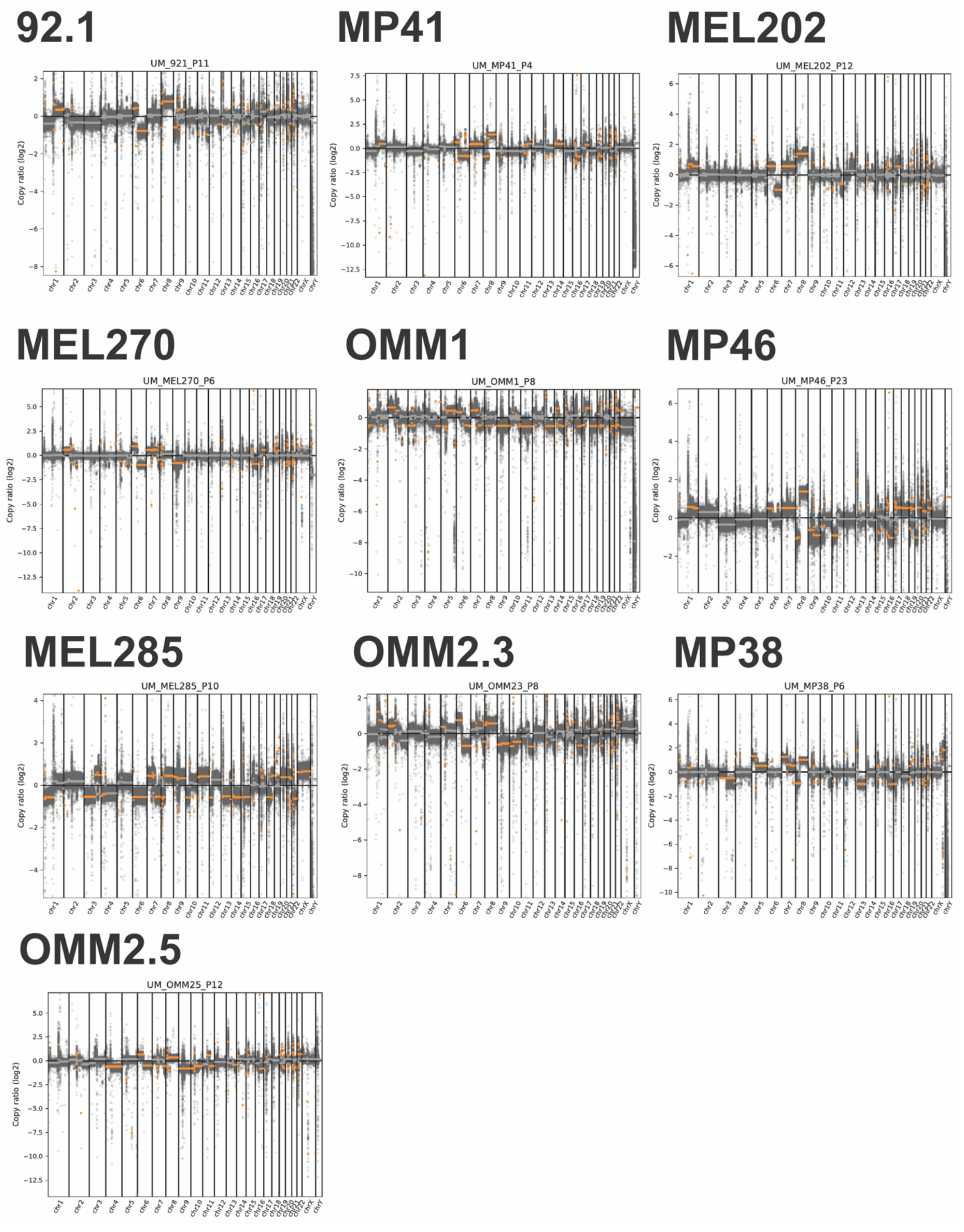


**Supplementary Fig. 2. Large-scale copy number profiles of uveal melanoma cell lines.**
These profiles were generated using CNVkit on the whole genome sequences as described in the Methods. Calls are available in the Github and Figshare repositories associated with this paper.

a

| Chromosome | Position | Reference | Al | Cell Line | Gene | Amino Acid | Transcript ID |
|---|---|---|---|---|---|---|---|
| chr17 | 7676046 | C | A | UM_921_P11 | *TP53* | p.G69V | NM_001126118 |
| chr17 | 7676046 | C | A | UM_921_P11 | *TP53* | p.G108V | NM_000546 |
| chr19 | 3118944 | A | T | UM_MP41_P4 | *GNA11* | p.Q209L | NM_002067 |
| chr19 | 3118944 | A | T | UM_OMM1_P8 | *GNA11* | p.Q209L | NM_002067 |
| chr2 | 197402760 | G | C | UM_MEL202_P12 | *SF3B1* | p.R625G | NM_012433 |
| chr3 | 52409603 | TGACACCTGCGATGA | T | UM_MP38_P6 | *BAP1* | p.G23Efs*44 | NM_004656 |
| chr9 | 77794569 | C | T | UM_MEL202_P12 | *GNAQ* | p.R210K | NM_002072 |
| chr9 | 77794572 | T | A | UM_921_P11 | *GNAQ* | p.Q209L | NM_002072 |
| chr9 | 77794572 | T | A | UM_MEL202_P12 | *GNAQ* | p.Q209L | NM_002072 |
| chr9 | 77794572 | T | G | UM_MEL270_P6 | *GNAQ* | p.Q209P | NM_002072 |
| chr9 | 77794572 | T | G | UM_MP38_P6 | *GNAQ* | p.Q209P | NM_002072 |
| chr9 | 77794572 | T | A | UM_MP46_P23 | *GNAQ* | p.Q209L | NM_002072 |
| chr9 | 77794572 | T | G | UM_OMM23_P8 | *GNAQ* | p.Q209P | NM_002072 |
| chr9 | 77794572 | T | G | UM_OMM25_P12 | *GNAQ* | p.Q209P | NM_002072 |
| chrX | 20138622 | C | T | UM_921_P11 | *EIF1AX* | p.G6D | NM_001412 |

b

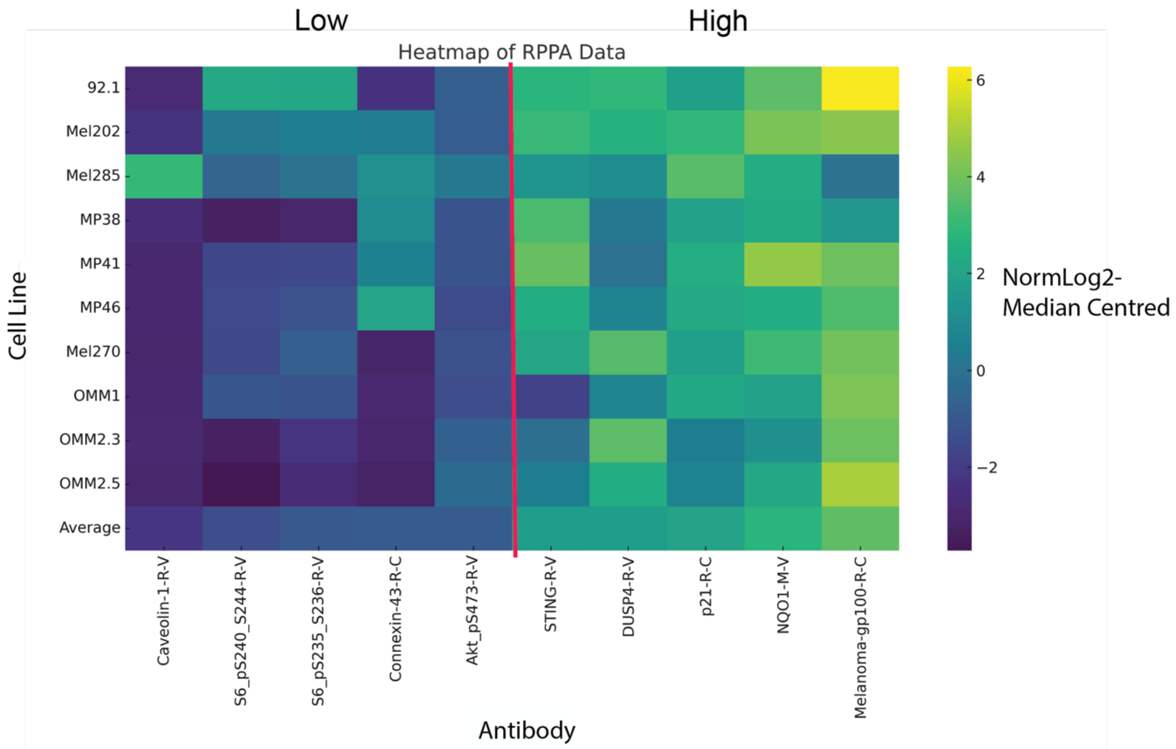


**Supplementary Fig. 3. Uveal melanoma cell line characterisation.** (a) Variants in established uveal melanoma drivers. (b). Proteins with the highest and lowest normalised $Log_2$ median centred values as determined by reverse-phase protein array (RPPA) analysis[1]. See Methods. Supplementary Table 2 contains the raw data.

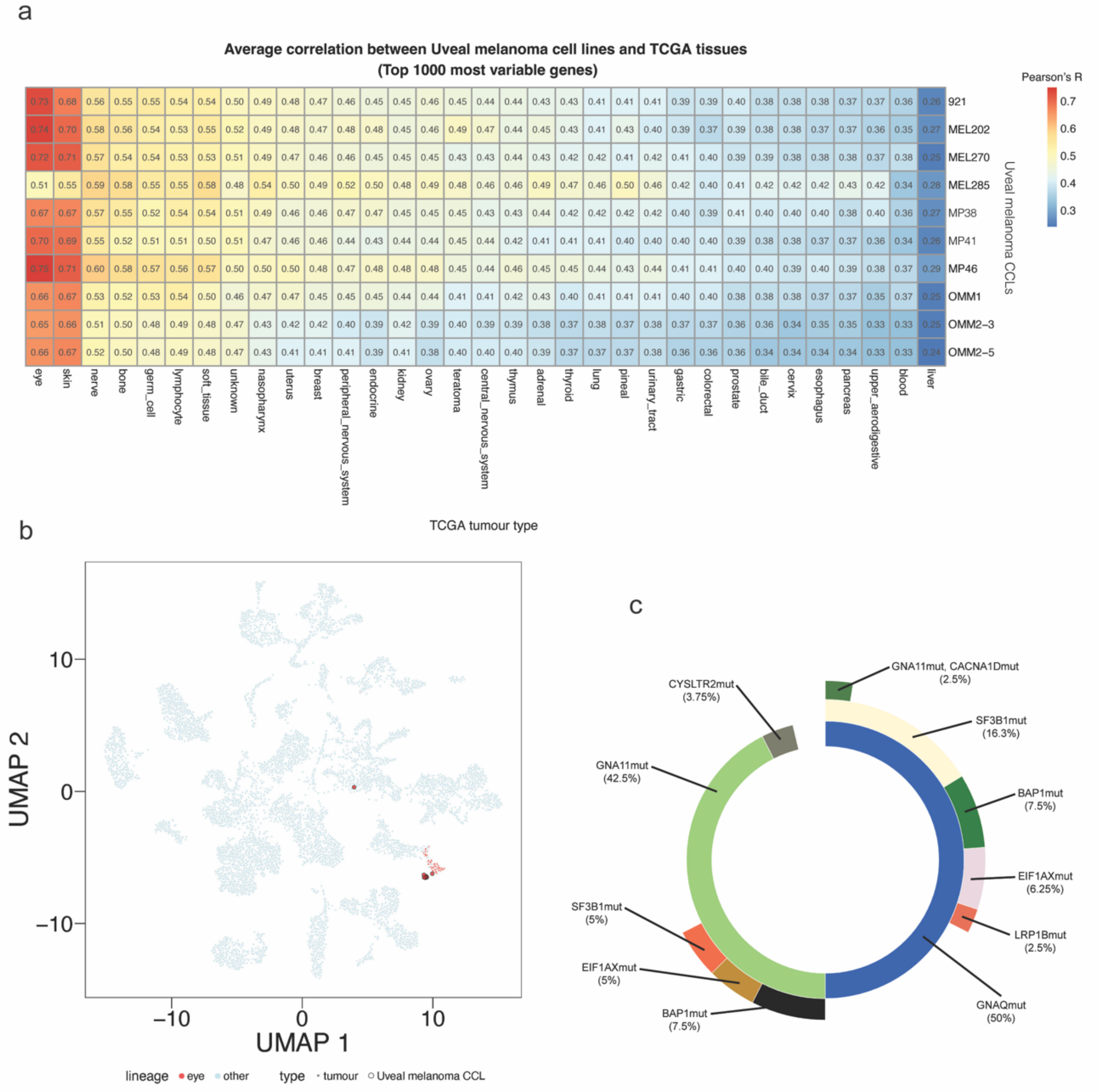


d

| Tumour.SubType.Index | | Representative.Cell.Line Mutant Gene | Percentage.patients |
|---|---|---|---|
| 1 | OMM23 | GNAQmut | 50 |
| 2 | MP41 | ~GNAQmut, GNA11mut | 42.5 |
| 3 | MEL202 | GNAQmut, SF3B1mut | 16.25 |
| 4 | MP38 | GNAQmut, ~SF3B1mut, BAP1mut | 7.5 |
| 6 | 921 | GNAQmut, ~SF3B1mut, ~BAP1mut, EIF1AXmut | 6.25 |
| 7 | MP46 | GNAQmut, ~SF3B1mut, ~BAP1mut, ~EIF1AXmut, LRP1Bmut | 2.5 |
| 1 | OMM25 | GNAQmut | 50 |
| 2 | OMM1 | ~GNAQmut, GNA11mut | 42.5 |
| 3 | MEL270 | GNAQmut, SF3B1mut | 16.25 |

'~'without.

**Supplementary Fig 4. Alignment of uveal melanoma cell lines with TCGA uveal melanoma genome and transcriptome data.** (a) Shown is the Pearson correlation between the 10 uveal melanoma lines and TCGA uveal (and other) tumors based on RNA-seq data. (b) Uniform Manifold Approximation and Projection (UMAP) of expression data. These comparisons were generated using Celligner[2]. The small red dots are uveal tumors and the larger red circles cell lines. All cell lines very tightly clustered with uveal melanoma, except for MEL285, a monosomy 3 line, which has highest similarity to eye and skin tumors (see above). (c-d) Analysis of the 10 uveal melanoma lines and TCGA uveal melanomas based on genomic features. This analysis suggests our collection captures all major uveal melanoma drivers. This analysis was performed using CELLector[3] (see Methods).

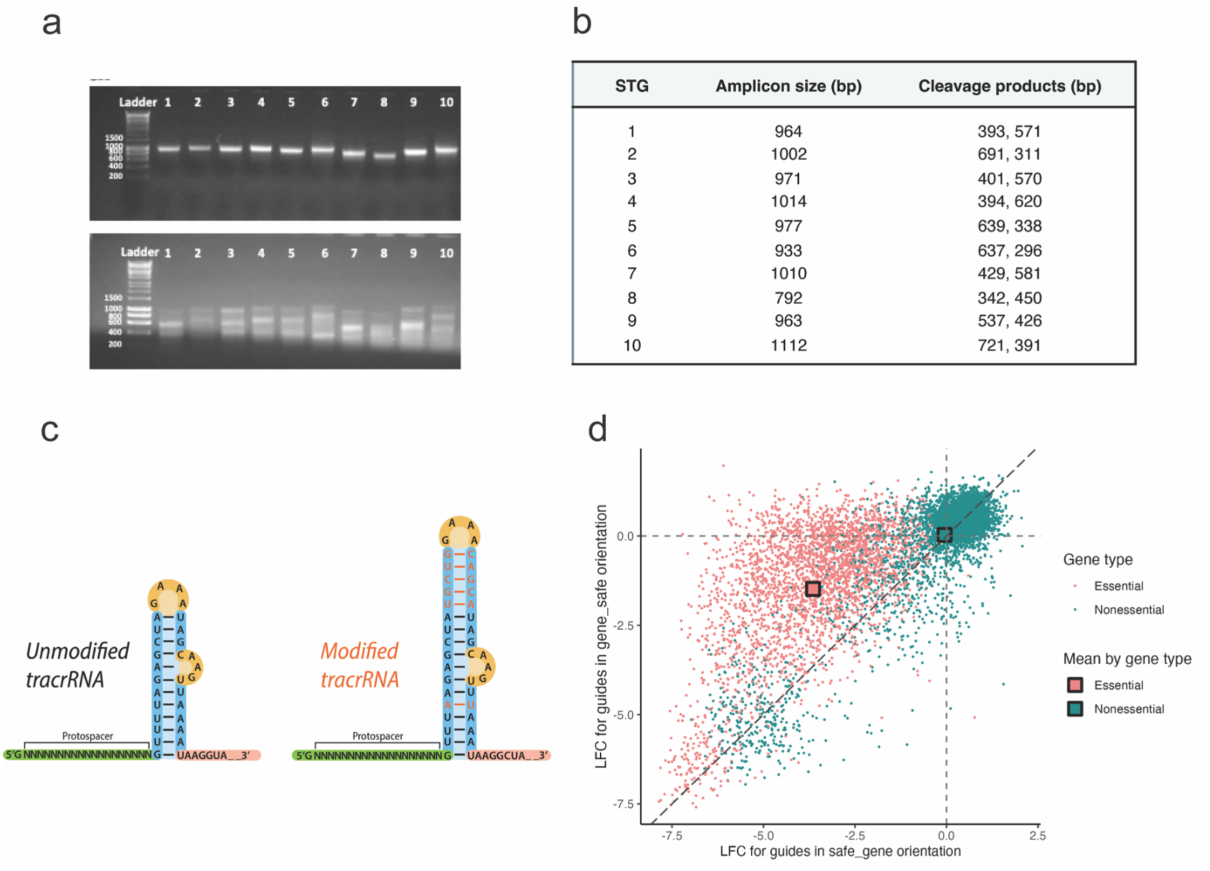


| STG | Amplicon size (bp) | Cleavage products (bp) |
|---|---|---|
| 1 | 964 | 393, 571 |
| 2 | 1002 | 691, 311 |
| 3 | 971 | 401, 570 |
| 4 | 1014 | 394, 620 |
| 5 | 977 | 639, 338 |
| 6 | 933 | 637, 296 |
| 7 | 1010 | 429, 581 |
| 8 | 792 | 342, 450 |
| 9 | 963 | 537, 426 |
| 10 | 1112 | 721, 391 |

**Supplementary Fig 5. Validation of safe-targeting guides and tracrRNA sequences.**
(a) Gel electrophoresis indicating indel formation in A375-Cas9 cells that have been transduced with lentiviruses expressing safe targeting guides (STGs) 1 to 10 (corresponding lanes 1 to 10)[4]. The top image shows the amplicon size prior to nuclease digestion. The bottom image the cleavage product after treatment with the Surveyor nuclease. This experiment was performed once. (b) Initial amplicon sizes and resulting cleavage product sizes for each STG. These experiments were performed as outlined in Methods. (c) Unmodified and modified tracrRNA used in the vector. We provide the sequence and annotation of the vector in Figshare. (d) Y-axis are gRNAs behind the hU6 promoter. X-axis are gRNAs behind the mU6 promoter. The balanced design with guides in both positions was used to mitigate differences in promoter strengths with the hU6 promoter “stronger” in this analysis.

a

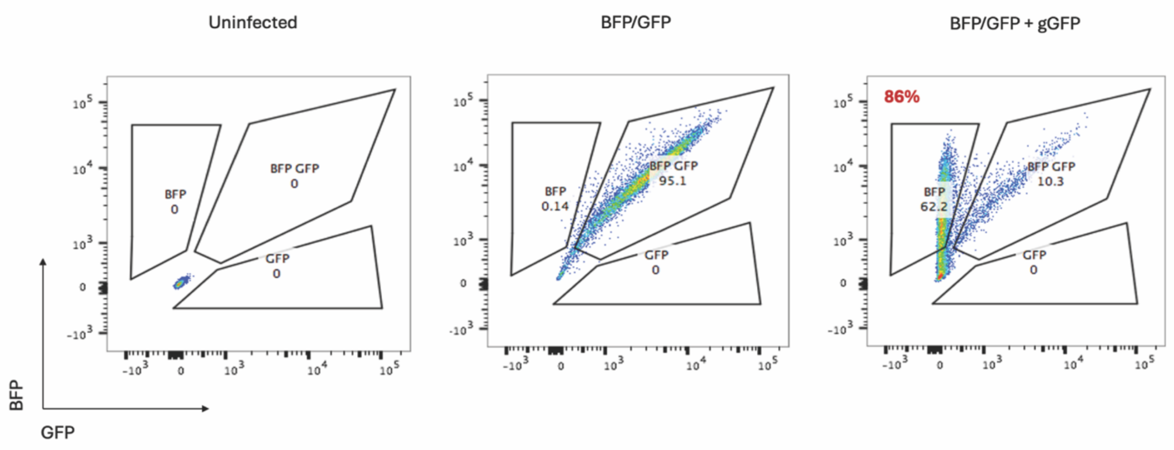


b

| Cell line (% Cas9 activity) | |
|---|---|
| 92.1 | (92.9) |
| Mel202 | (98.1) |
| Mel270 | (90.7) |
| Mel285 | (93.6) |
| MP38 | (97.9) |
| MP41 | (85.8) |
| MP46 | (94.5) |
| OMM1 | (94.8) |
| OMM2.3 | (99.0) |
| OMM2.5 | (96.8) |

**Supplementary Fig. 6. Assessment of Cas9 activity in uveal melanoma cell lines.** (a) Flow cytometry dot plots showing representative fluorescent expression patterns/gating for confirmation of Cas9 activity (the assay was performed after each line was derived and before each CRISPR screen). Cas9 activity was quantified by determining the proportion of BFP+/GFP- cells (right plot) within the BFP+ cell population. All ten uveal melanoma cell lines had Cas9 activity quantified using this method. Cell line MP41, with a Cas9 activity of 86% is shown, and is representative of the Cas9 expression of the other cell lines. gGFP; guide RNA targeting the GFP coding sequence. (b) Percentage Cas9 activity for all screened cell lines.

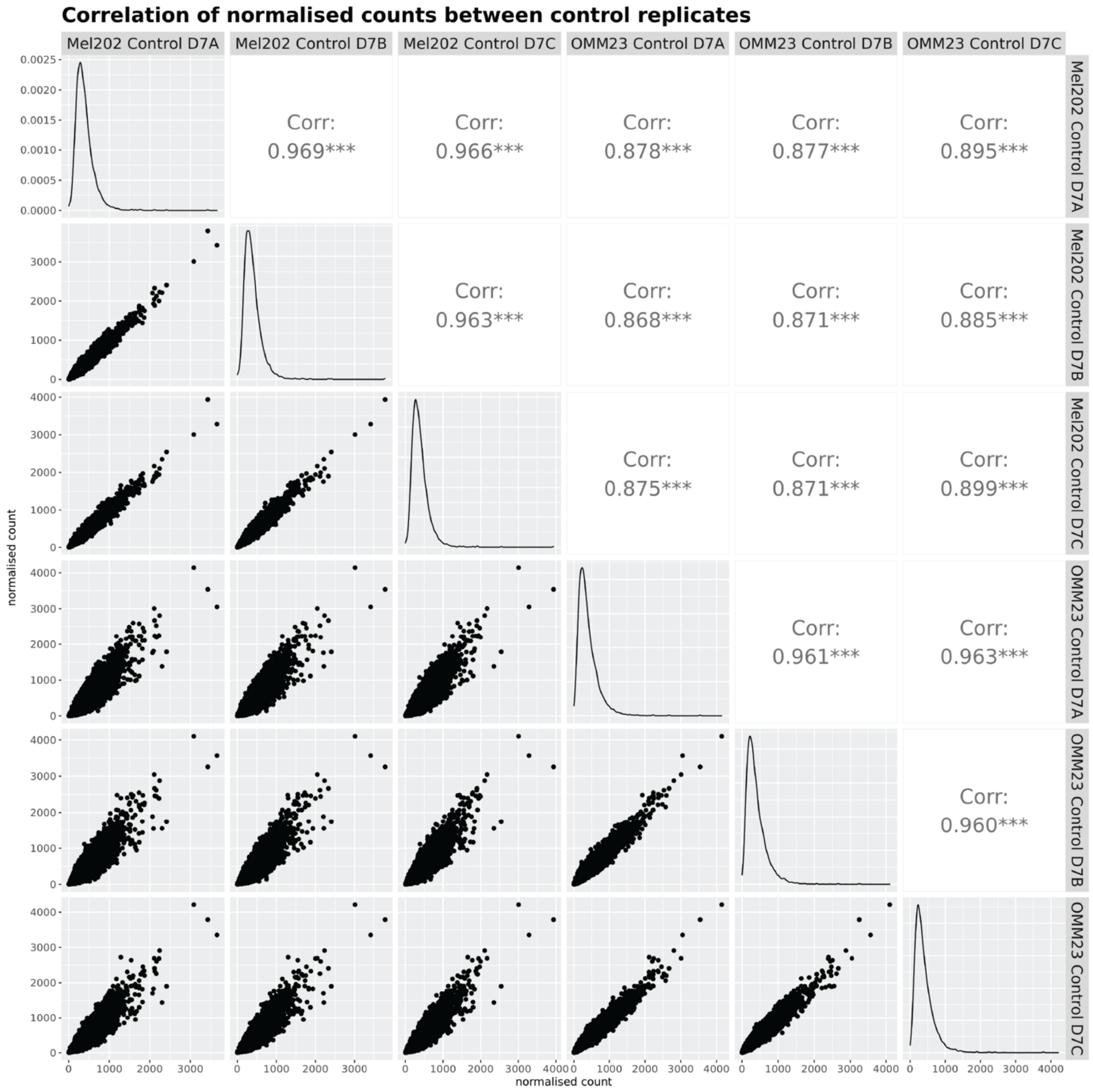


**Supplementary Fig. 7. Spearman's correlation of normalised counts of guides in each replicate from the two Cas9 negative control lines (MEL202 and OMM2.3) and the mean of these controls.**

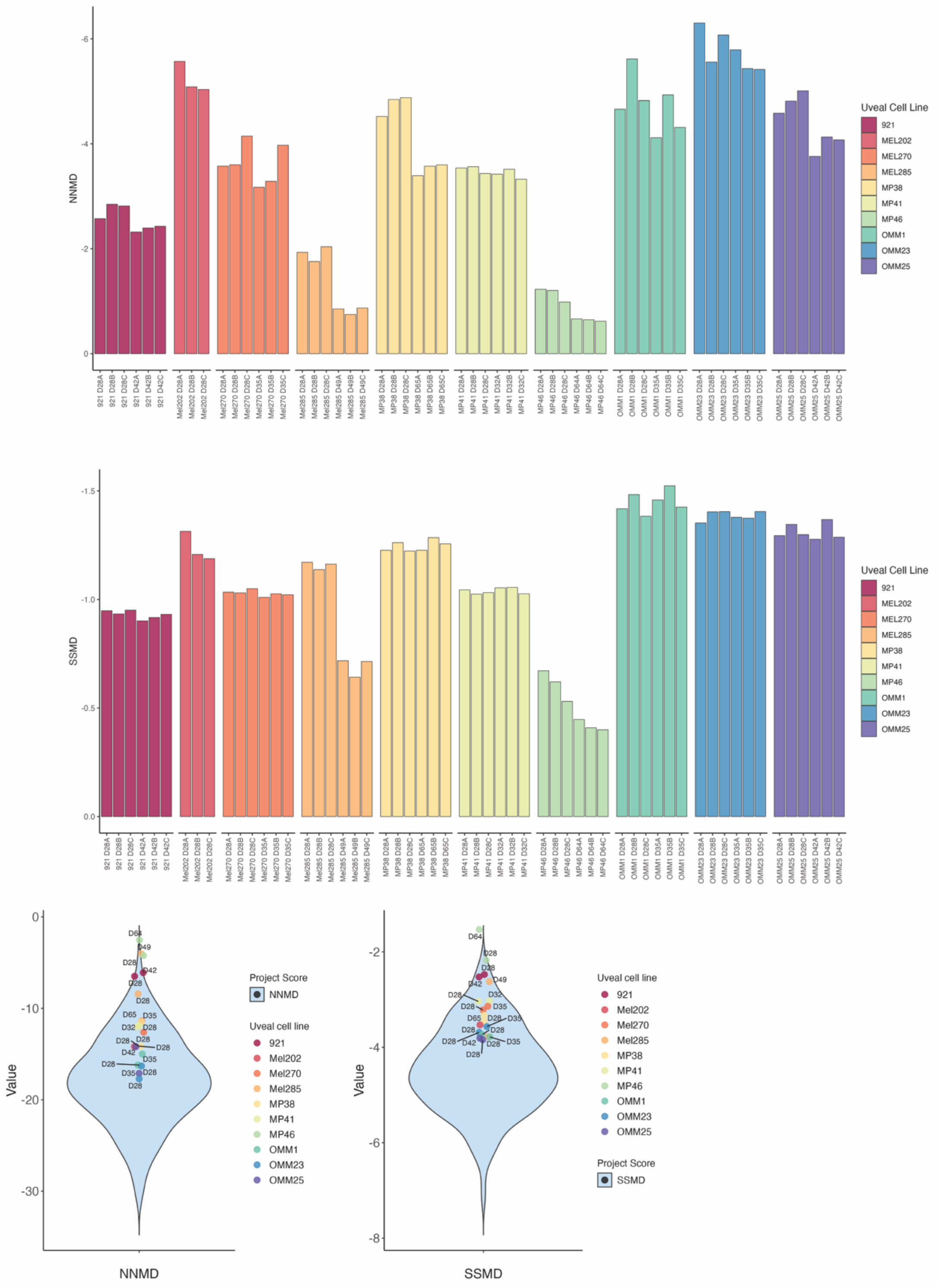


**Supplementary Fig. 8. Uveal melanoma combinatorial screen essential and non-essential gene separation metrics.** NNMDs (Null normalised mean differences) derived from guide-level log-fold changes (LFCs) of essential and non-essential control genes in each screen replicate (top). SSMDs (Strictly standardised mean differences) derived from guide-level LFCs of essential and non-essential control genes in each screen replicate (middle). NNMD/SSMDs[5] derived from mean gene-level LFCs of essential and non-essential control genes in each cell line, compared to Project Score screens (bottom). The data is provided for each timepoint. D; day. Differences in the NNMDs for Project Score screens to the whole genome data can be accounted for by re-computing NNMDs using the subset of essential and non-essential control genes in the combinatorial library.

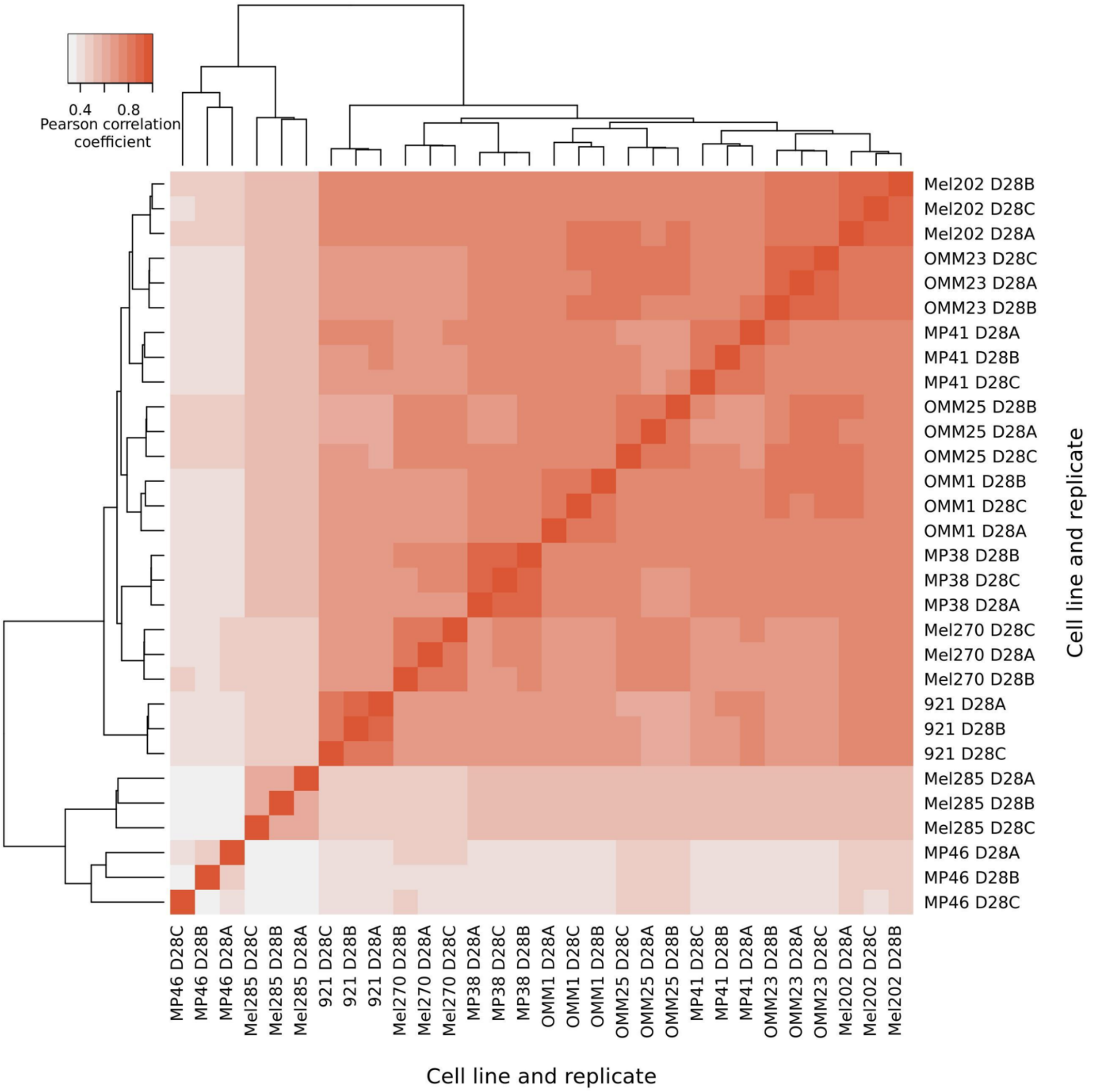


**Supplementary Fig. 9. Assessment of combinatorial CRISPR screen replicate correlations.** Pearson correlation heatmap of raw $\log_2$ fold changes between all pairwise combinations for each replicate across all 10 cell lines that were included in the combinatorial CRISPR screen.

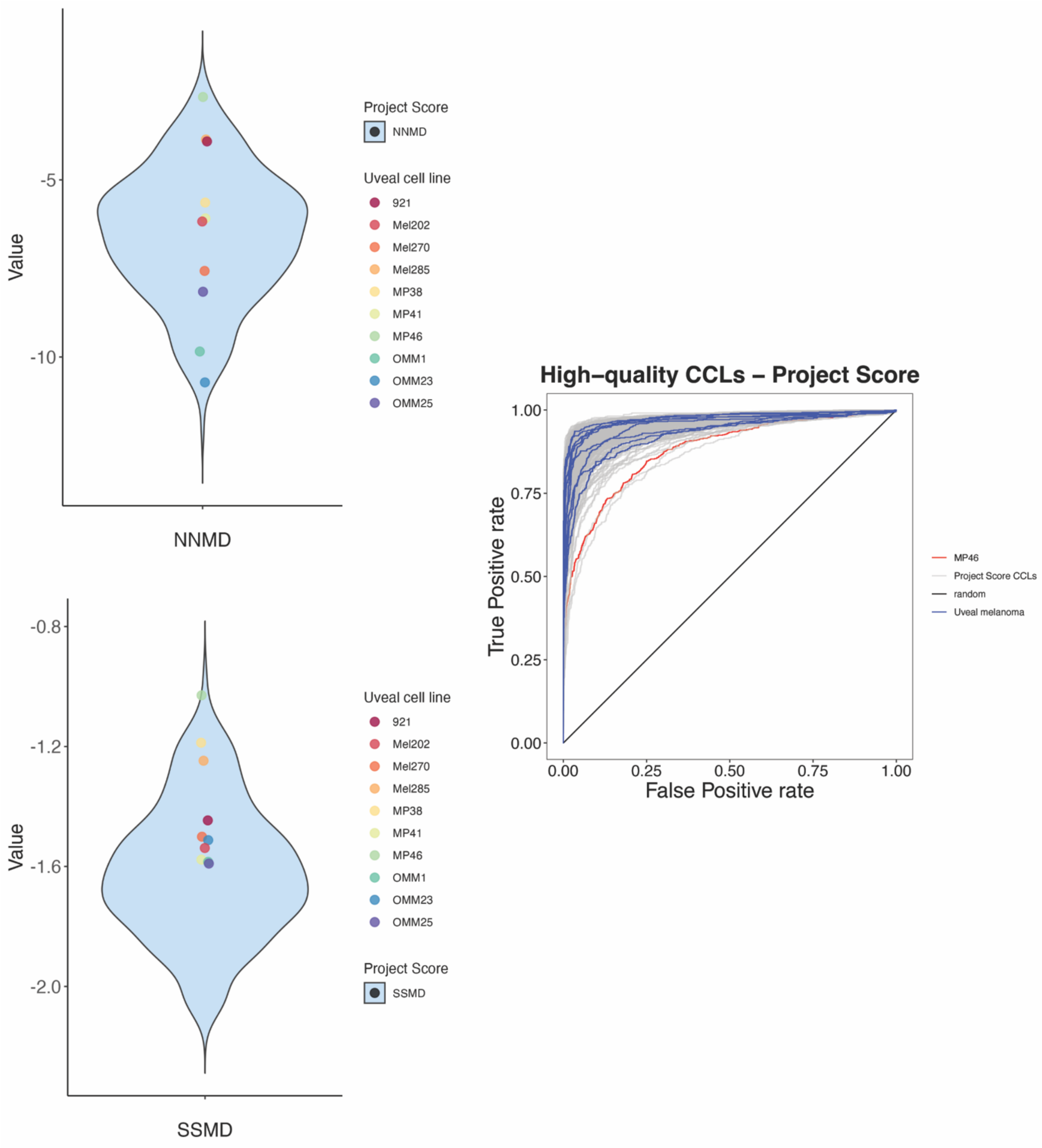


**Supplementary Fig. 10. Assessment of genome-wide sgRNA CRISPR knockout screen performance relative to Project Score data (see Methods).** NNMDs (Null normalised mean differences) and SSMDs (Strictly standardised mean differences). This analysis reveals that all screens were of high quality passing the Project Score/DepMap screen metrics[6]. The NNMD figure shown is the same as in Fig. 3 and reproduced here for completeness/comparison.

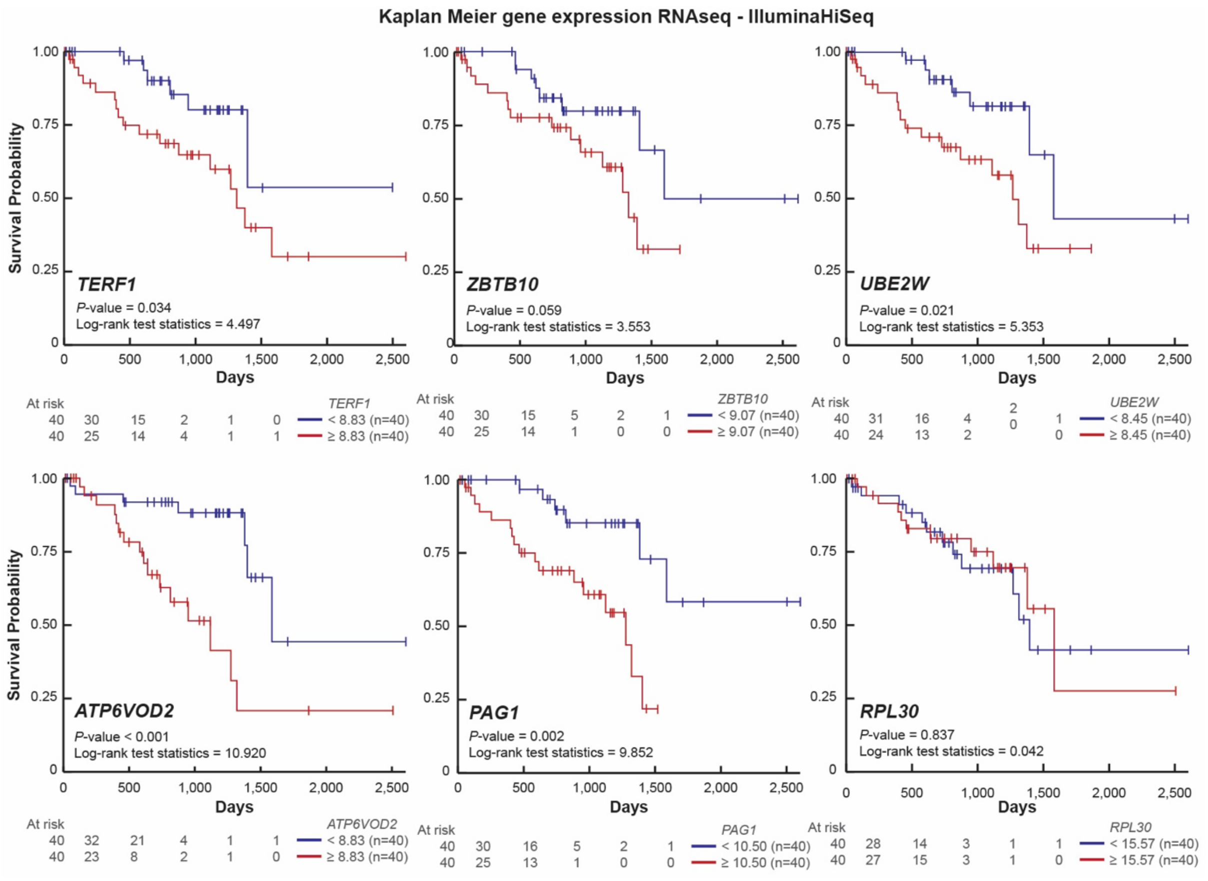


**Supplementary Fig. 11. Selected essentialities associated with genomic amplifications/copy number events.** In this analysis we identified recurrent genetic changes in our uveal melanoma cell line collection (copy number >5; $Log_2$ >1.322) and then defined which of the genes in these regions are amongst the 76 genes we identified as uveal melanoma-specific/essential. We further examined these genes to determine the effect of gene expression on patient outcome (overall survival) using gene expression data from the TCGA uveal melanoma project; Robertson et al., 2017[7]. Values derived from RSEM.

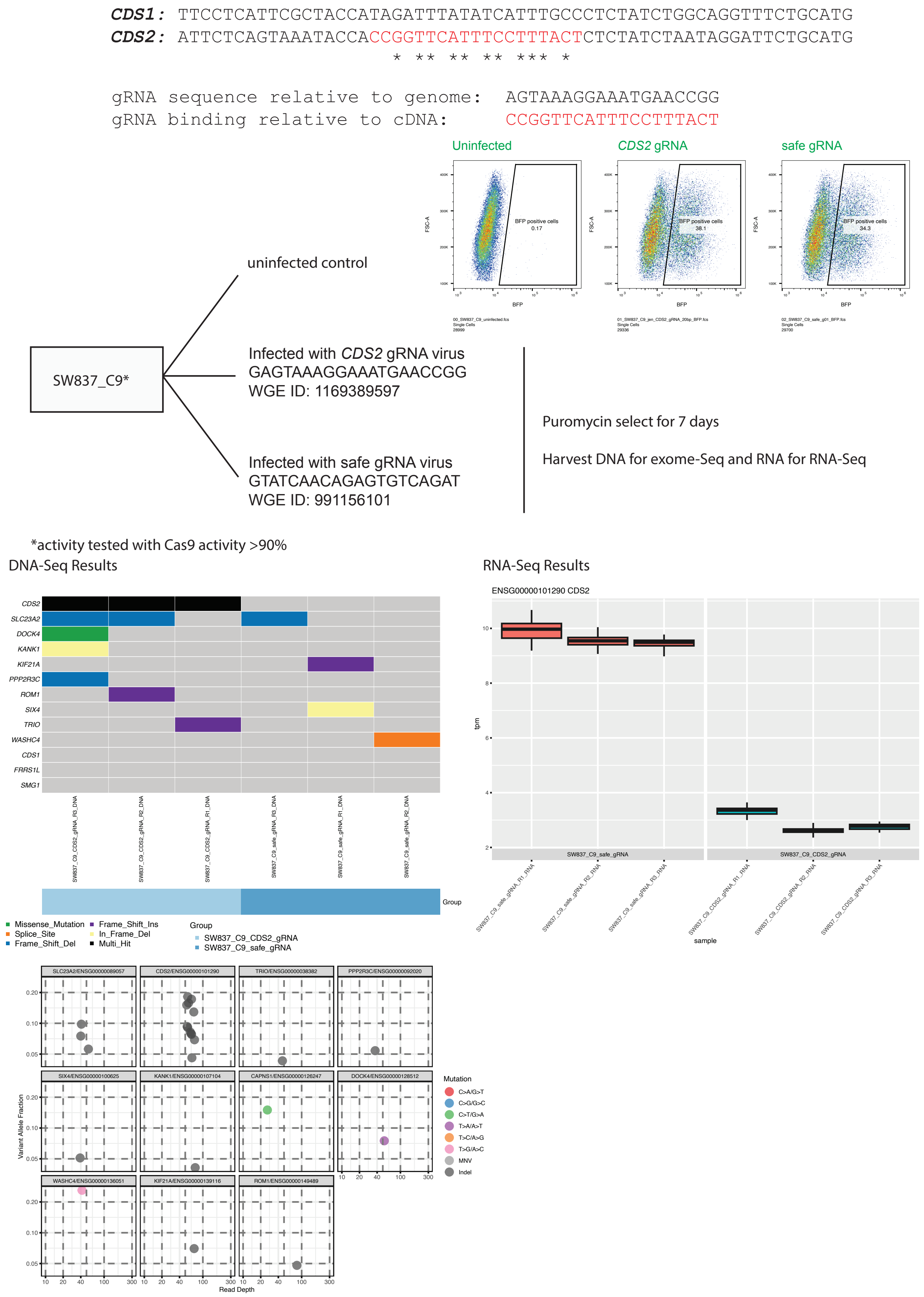


**Supplementary Fig. 12. Analysis of on/off-target cutting with the *CDS2* and STG1 gRNAs in SW837 cells.** The experimental design is shown with a FACS plot illustrating the transduction efficiency

of cells with lentiviral vectors carrying *CDS2* (middle) or *STG1* (right) gRNAs prior to 7 days of selection in puromycin. Uninfected (Cas9-expressing SW837_C9) cells were used as a control (left). The *CDS2* gRNA generated multiple disruptive indel events. The tile plot shows a summary variants found by somatic callers in targeted lines using the SW837_C9 un-transfected control as a normal/reference sample. The left 3 samples were from SW837_C9 cells transfected with a *CDS2* gRNA. Right 3 samples were from SW837_C9 cells transfected with the *STG1* gRNA. Non-recurrent variants in five genes (*DOCK4, KANK1, PPP2R3C, ROM1, & TRIO*), which might be the result of off-target cutting (although not at predicted sites) or could be cell culture artefacts, were also observed. No off-target was observed on *CDS1* or the two potential predicted exonic off-target sites for *CDS2* gRNA on *FRRS1L* and *SMG1*. Potential off-target *STG1* gRNA or cell culture artefact events in the (*WASHC4, SIX4*, *KIF21A*) genes was found. Of note, a recurrent 1bp deletion on *SLC23A2* was found in two *CDS2* targeted replicates and one *STG1.* Since the *STG1* gRNA cutting site is not in an exon we did not detect cutting with this gRNA. Transcriptome sequence analysis was performed. This revealed, as expected, disruption of *CDS2* transcripts which were significantly differentially expressed; qval: 2.99009556852052e-80. Log$_2$FC -1.77 (See Methods). For brevity we have not provided the other 9 differentially expressed protein coding genes in this analysis but provide them in Github. The data accession number is: ERP159012. Following these results, we used these highly specific and active gRNAs for all validation experiments.

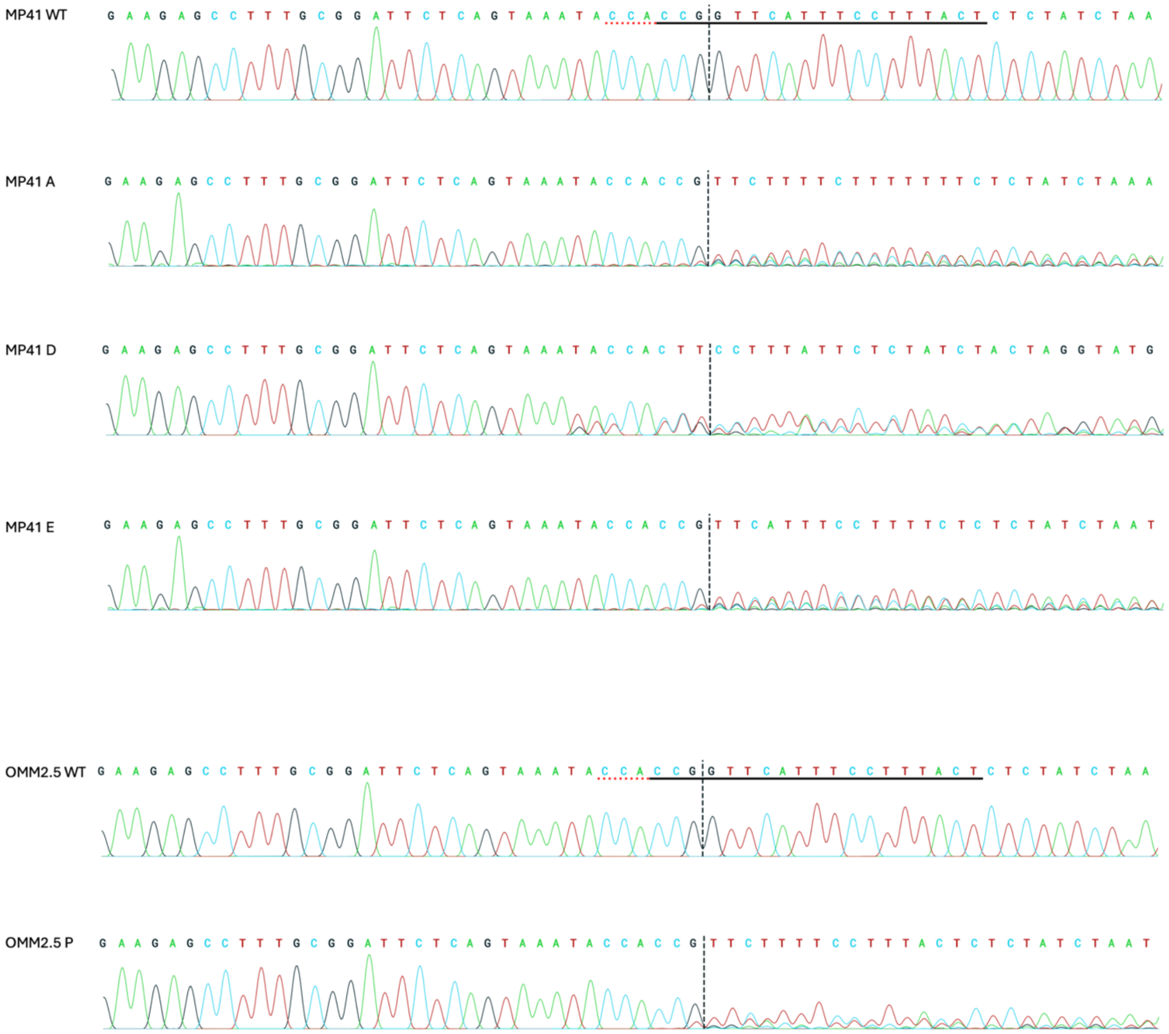


**Supplementary Fig. 13. Generation of isogenic doxycycline-inducible *CDS2* knockout cell lines.** Sanger sequencing of *CDS2* knockout clones from the cell lines MP41 and OMM2.5. The *CDS2* gRNA sequence is underlined in the wildtype cell lines. These data show efficient gRNA-mediated gene disruption and represent PCR sequencing of the targeted locus amplified from genomic DNA.

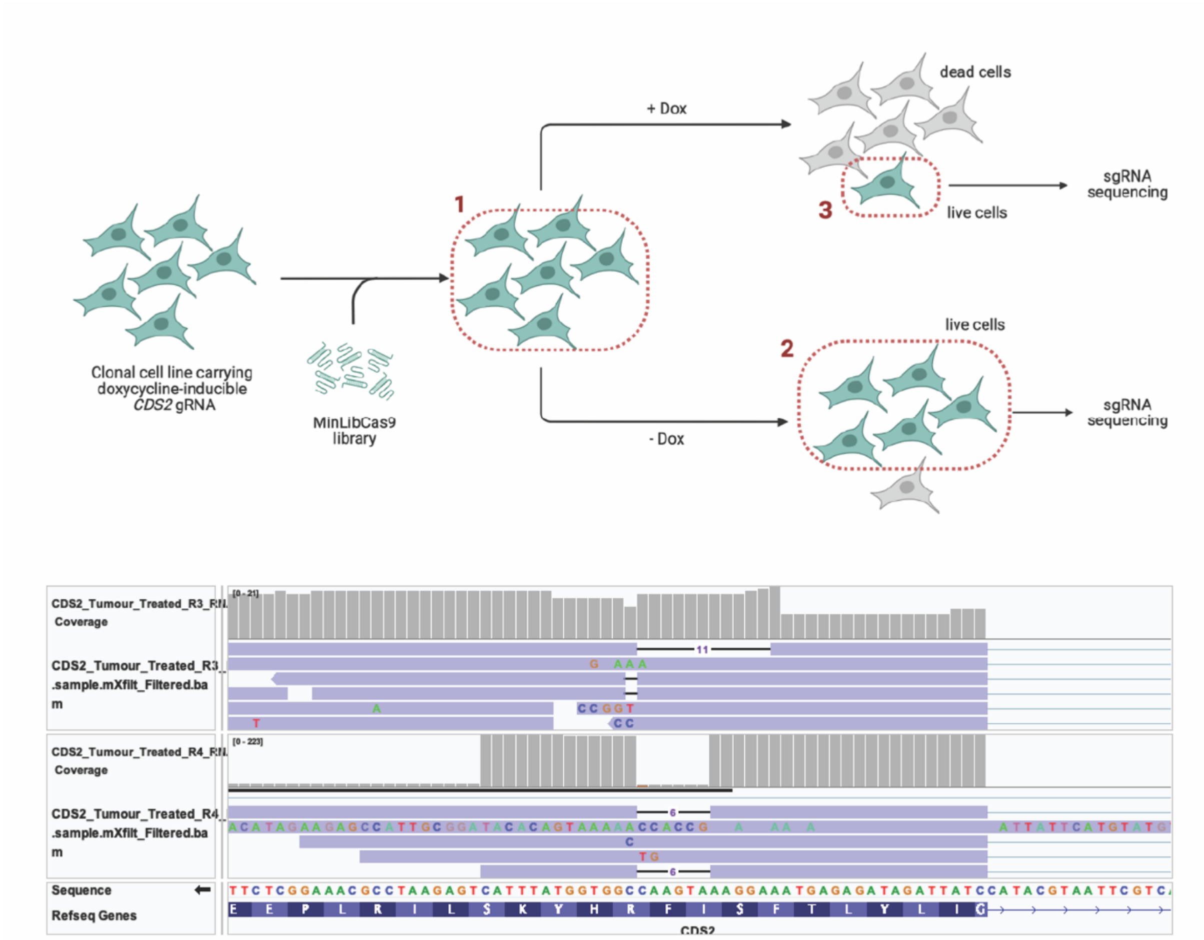


**Region shown: chr20:5,178,894-5,178,971**
**gRNA binding site: 20:5178922-5178944 Strand -**
***CDS2* gRNA –GAGTAAAGGAAATGAACCGG**

**Supplementary Fig. 14. Schematic showing the design of the genetic suppressor/rescue screen and results of xenograft transcriptome sequencing.** A clonal population of doxycycline-inducible MP41 *CDS2* knockout cells underwent genome-wide CRISPR knockout screening using the MinLibCas9 library[8] (top). Following puromycin selection, half of the cells received doxycycline for the duration of the screen, to induce *CDS2* disruption, and the remaining half were treated with the equivalent volume of DMSO. After 21 days, the surviving cells in each population were harvested. Genomic DNA was extracted and underwent library preparation for sequencing of the single gRNA region to determine whether any enriched genes were present within the doxycycline-treated cohort that resulted in cell rescue following *CDS2* loss. Cells marked with dotted red lines were sequenced (1, pre-doxycycline samples; 2, DMSO-treated samples, 3, doxycycline-treated samples). To further explore possible mechanisms of resistance to *CDS2* disruption we collected, and transcriptome sequenced residual uveal melanoma cells from xenografted mice. This involved using human exome capture baits (to capture cDNA) with reads subsequent filtered using XenofilteR[9]. This analysis is provided in the Github. Although we were unable to find a unifying mechanism of resistance, we could identify non-disruptive edits at the *CDS2* target RNA (bottom), including in-frame deletions. Two examples are shown; The top example shows residual wildtype reads and a 6bp inframe deletion. The bottom example shows an edited read that is likely not disruptive amongst other reads that are frameshifts. The depth over the region was >50x so only a subset of reads are shown.

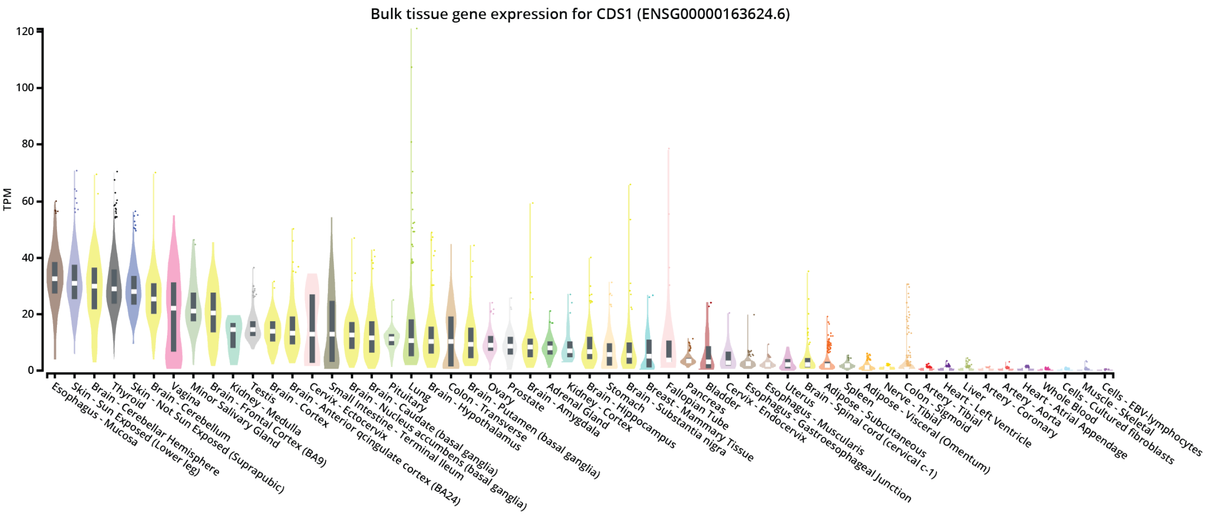


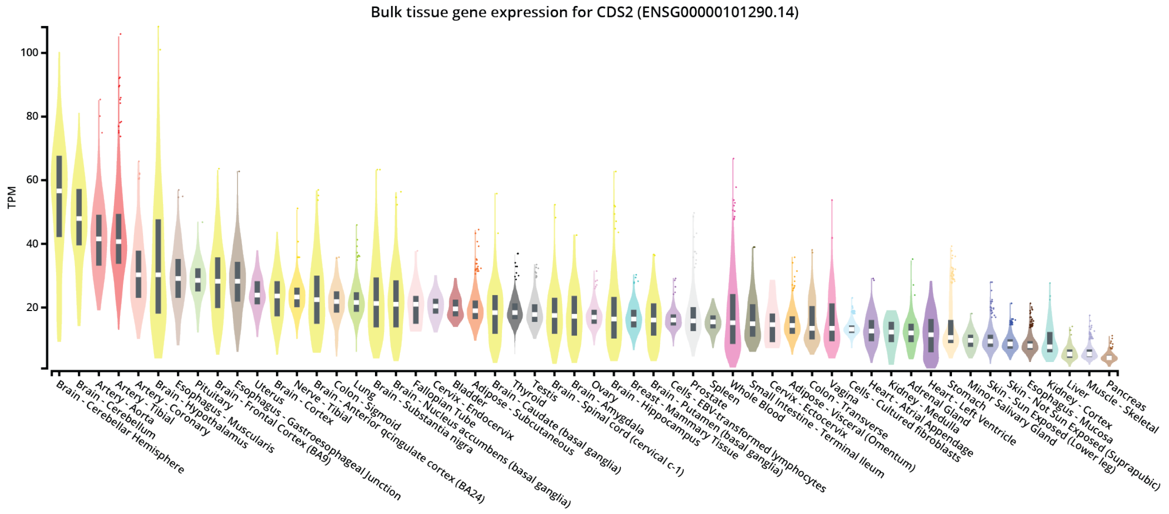


**Supplementary Fig. 15. GTex[10] normal tissue expression data for *CDS1* and *CDS2*.** Data downloaded for analysis on the 1st March 2025. Expression values are Transcripts Per Million (TPM) calculated from gene models with isoforms collapsed to a single gene. No normalisation has been applied. Box plots are shown as median and 25th and 75th percentiles; points are displayed as outliers if they are 1.5 times the interquartile range.

## References


1. Chen, M.M. *et al.* TCPA v3.0: An Integrative Platform to Explore the Pan-Cancer Analysis of Functional Proteomic Data. *Mol Cell Proteomics* **18**, S15-s25 (2019).
2. Warren, A. *et al.* Global computational alignment of tumor and cell line transcriptional profiles. *Nat Commun* **12**, 22 (2021).
3. Najgebauer, H. *et al.* CELLector: Genomics-Guided Selection of Cancer In Vitro Models. *Cell Syst* **10**, 424-432.e6 (2020).
4. Morgens, D.W. *et al.* Genome-scale measurement of off-target activity using Cas9 toxicity in high-throughput screens. *Nat Commun* **8**, 15178 (2017).
5. Dempster, J.M. *et al.* Chronos: a cell population dynamics model of CRISPR experiments that improves inference of gene fitness effects. *Genome Biol* **22**, 343 (2021).
6. Behan, F.M. *et al.* Prioritization of cancer therapeutic targets using CRISPR-Cas9 screens. *Nature* **568**, 511-516 (2019).
7. Robertson, A.G. *et al.* Integrative Analysis Identifies Four Molecular and Clinical Subsets in Uveal Melanoma. *Cancer Cell* **32**, 204-220 e15 (2017).
8. Goncalves, E. *et al.* Minimal genome-wide human CRISPR-Cas9 library. *Genome Biol* **22**, 40 (2021).
9. Kluin, R.J.C. *et al.* XenofilteR: computational deconvolution of mouse and human reads in tumor xenograft sequence data. *BMC Bioinformatics* **19**, 366 (2018).
10. The Genotype-Tissue Expression (GTEx) project. *Nat Genet* **45**, 580-5 (2013).
