# Supplementary material for: The synthetic lethal interaction between CDS1 and CDS2 is a vulnerability in uveal melanoma and across multiple tumor types
Source: Nat Genet. 2025 Jul 4;57(7):1672–83. doi: 10.1038/s41588-025-02222-1 (PMC12283370; doi:10.1038/s41588-025-02222-1)
Supplement: Supplementary file 2 — Reporting Summary [file 41588_2025_2222_MOESM2_ESM.pdf]

nature portfolio

Corresponding author(s): Adams DJ: LE64078R

Last updated by author(s): 30th March 2025

Reporting Summary

Nature Portfolio wishes to improve the reproducibility of the work that we publish. This form provides structure for consistency and transparency in reporting. For further information on Nature Portfolio policies, see our Editorial Policies and the Editorial Policy Checklist.

Statistics

For all statistical analyses, confirm that the following items are present in the figure legend, table legend, main text, or Methods section.

n/a	Confirmed	
☐	☒	The exact sample size (*n*) for each experimental group/condition, given as a discrete number and unit of measurement
☐	☒	A statement on whether measurements were taken from distinct samples or whether the same sample was measured repeatedly
☐	☒	The statistical test(s) used AND whether they are one- or two-sided<br>*Only common tests should be described solely by name; describe more complex techniques in the Methods section.*
☐	☒	A description of all covariates tested
☐	☒	A description of any assumptions or corrections, such as tests of normality and adjustment for multiple comparisons
☐	☒	A full description of the statistical parameters including central tendency (e.g. means) or other basic estimates (e.g. regression coefficient) AND variation (e.g. standard deviation) or associated estimates of uncertainty (e.g. confidence intervals)
☐	☒	For null hypothesis testing, the test statistic (e.g. *F*, *t*, *r*) with confidence intervals, effect sizes, degrees of freedom and *P* value noted<br>*Give P values as exact values whenever suitable.*
☒	☐	For Bayesian analysis, information on the choice of priors and Markov chain Monte Carlo settings
☒	☐	For hierarchical and complex designs, identification of the appropriate level for tests and full reporting of outcomes
☐	☒	Estimates of effect sizes (e.g. Cohen's *d*, Pearson's *r*), indicating how they were calculated

Our web collection on statistics for biologists contains articles on many of the points above.

Software and code

Policy information about availability of computer code

Data collection

No software was used for data collection, except for proprietary Illumina base-calling software on HiSeq platforms.

Data analysis

SAMtools v1.10 was used to convert BAM files to FASTQ files. QC was performed using FastQC v0.11.8. Reads were aligned to GRCh38 using BWA mem v0.7.17. GATK v4.4.0.0 was used for germline short variant calling (SNVs, indels) with the HaplotypeCaller.

For transcriptome data - Reads were mapped using STAR v2.5.0c against the GRCh38 human reference genome with the ERCC spike-in sequences and ENSEMBL v103 gene annotation. To assess gene expression reads were counted using HTSeq-count v0.7.2 .XenofilteR after mapping with the STAR aligner. Expression assessment used Kallisto (0.51.1) and Sleuth (0.30.1).

CRISPR analysis
All samples were processed using CRISPRcleanR v3.0.159
C-SAR v1.3.6 (https://github.com/cancerit/C-SAR)
MAGeCK MLE v0.5.9.5

Single cell analysis
GBmap pipeline approach from Ruiz-Moreno et al.
STARsolo v2.7.10a pipeline
semi-supervised neural network model called single-cell ANnotation using Variational Inference (scANVI), within the transfer-learning framework of the single-cell architectural surgery algorithm (scArches).

Mass-spec

Proteome Discoverer 3.0 (Thermo Scientific) was used with SequestHT and Comet search engines for protein identification and quantification. Peptide confidence was estimated using Percolator, maintaining a false discovery rate (FDR) of 0.01 with target-decoy database validation.

Custom Code is available here: https://github.com/team113sanger/Targeting-the-CDS1-2-axis-as-a-therapeutic-strategy-in-uveal-melanoma-and-pan-cancer

For manuscripts utilizing custom algorithms or software that are central to the research but not yet described in published literature, software must be made available to editors and reviewers. We strongly encourage code deposition in a community repository (e.g. GitHub). See the Nature Portfolio guidelines for submitting code & software for further information.

Data

Policy information about availability of data

All manuscripts must include a data availability statement. This statement should provide the following information, where applicable:

- Accession codes, unique identifiers, or web links for publicly available datasets
- A description of any restrictions on data availability
- For clinical datasets or third party data, please ensure that the statement adheres to our policy

Code Availability Statement

Github:

CRISPR Screen Analysis: https://github.com/team113sanger/Targeting-the-CDS1-2-axis-as-a-therapeutic-strategy-in-uveal-melanoma-and-pan-cancer

UVMap Data: https://github.com/jpark27/CDS1-2

All figure code is freely available for download: https://doi.org/10.5281/zenodo.15025721

Figshare: https://figshare.com/account/home#/projects/184459

Data Accessibility Statement
The sequencing data generated as part of this study is available using the following European Nucleotide Accession numbers:
ERP151504: Paired guide (combinatorial) uveal melanoma screen data.
ERP151444: Single guide (CRISPRko) uveal melanoma screen data.
ERP151445: Uveal melanoma suppressor screen.
ERP110320: Uveal melanoma cell line WGS.
ERP130186: Whole transcriptome sequencing of uveal melanoma cell lines.
ERP159012: Sequencing of SW837 cells following CDS2 gRNA transduction and selection. Transcriptome sequencing of mouse tumors.
ERP159013: Sequencing of SW837 cells following CDS2 gRNA transduction and selection.

Proteome data generated as part of this study is available via the PRIDE repository:
PXD053752: Targeting the CDS1/2 axis as a therapeutic strategy in uveal melanoma and pan-cancer.
TCGA data was downloaded from the Xena Browser: https://xenabrowser.net/
All other data is in the supplementary information.

Research involving human participants, their data, or biological material

Policy information about studies with human participants or human data. See also policy information about sex, gender (identity/presentation), and sexual orientation and race, ethnicity and racism.

Reporting on sex and gender	N/A
Reporting on race, ethnicity, or other socially relevant groupings	N/A
Population characteristics	N/A
Recruitment	N/A
Ethics oversight	N/A

Note that full information on the approval of the study protocol must also be provided in the manuscript.

Field-specific reporting

Please select the one below that is the best fit for your research. If you are not sure, read the appropriate sections before making your selection.

☒ Life sciences ☐ Behavioural & social sciences ☐ Ecological, evolutionary & environmental sciences

For a reference copy of the document with all sections, see nature.com/documents/nr-reporting-summary-flat.pdf

Life sciences study design

All studies must disclose on these points even when the disclosure is negative.

Sample size	No explicit sample size was decided upon a priori, rather we used standardized workflows for CRISPR screening and analysis. For example the CRISPR screening approach we used was that used by DepMAP/ProjectScore (PMID: 33712601) i.e. 3 replicates per cell line. For the mouse tumour growth experiments we defined significance using a Mixed-effects model with the Geisser-Greenhouse correction. With 16 mice per group and with at least 5 measurements per mouse/tumour we estimate power of ~70% to detect an effect of genotype on tumour growth. For all other experiments we either used all available data to make comparisons (for example all CRISPR screened cell lines) or independently replicated the experiments at least 3 times and an alpha value of 0.05.
Data exclusions	Only QC failed data was excluded and these parameters are clearly outlined in the paper. For example if NNMD values for CRISPR screens were not met or insufficient sequence data was generated.
Replication	All experiments where statistics were applied were repeated at least three times independently as indicated in the figure legend. This included separate days of transfection and distinct cell cultures. All replication attempts were successful.
Randomization	For the mouse experiments shown in figure 6 mice were randomised for tumour growth studies with an equal number of animals being assigned to each group. For CRISPR validation experiments gRNAs were randomly assigned to wells/cultures to avoid any biases. CRISPR screens were performed in pools with random virus infection so these experiments were randomised internally.
Blinding	Blinding was not performed but all key experiments were replicated by independent individuals in the lab. The CRISPR screens were performed en masse so there was no selection for screen outcomes.

Reporting for specific materials, systems and methods

We require information from authors about some types of materials, experimental systems and methods used in many studies. Here, indicate whether each material, system or method listed is relevant to your study. If you are not sure if a list item applies to your research, read the appropriate section before selecting a response.

Materials & experimental systems

n/a	Involved in the study	
☐	☒	Antibodies
☐	☒	Eukaryotic cell lines
☒	☐	Palaeontology and archaeology
☐	☒	Animals and other organisms
☒	☐	Clinical data
☒	☐	Dual use research of concern
☒	☐	Plants

Methods

n/a	Involved in the study	
☒	☐	ChIP-seq
☐	☒	Flow cytometry
☒	☐	MRI-based neuroimaging

Antibodies

Antibodies used	anti-CDS2 antibody (Proteintech 13175-1-AP, 1:1000 dilution) and an anti-vinculin antibody (ThermoFisher MA5-11690, 1:10,000 dilution)
Validation	The CDS2 antibody was validated by western blotting of KO cells. i.e. to show protein loss. The vinculin antobody has been extensively validated by the manufacturer using null cell lines (https://www.thermofisher.com/antibody/product/Vinculin-Antibody-clone-VLN01-Monoclonal/MA5-11690).

Eukaryotic cell lines

Policy information about cell lines and Sex and Gender in Research

Cell line source(s)	These are stated in Supplementary Table 1 and include ATCC, ECACC and the University of Liverpool Ocular melanoma tisseu bank.
Authentication	STR profiling was performed on all lines
Mycoplasma contamination	All lines were screened and found negative for mycoplasma. The were also routinely tested.
Commonly misidentified lines (See ICLAC register)	No commonly misidentified lines were used in this study.

Animals and other research organisms

Policy information about studies involving animals; ARRIVE guidelines recommended for reporting animal research, and Sex and Gender in Research

Laboratory animals	NOD-Prkdcscid-IL2rgTm1/Rj background. 8 weeks. Female mice. Mice were maintained in a specific pathogen-free unit on a 12h light:12h dark cycle. The ambient temperature is 21±2°C, and the humidity is 55±10%. Mice were housed using a stocking density of 3–5 mice per cage (overall dimensions of caging: 365×207×140mm3 (length×width×height), floor area 530cm2) in individually ventilated caging receiving 60 air changes per hour. In addition to Aspen bedding substrate, standard environmental enrichment of two Nestlets, a cardboard fun tunnel, and three wooden chew blocks are provided. Mice were given water and diet ad libitum.
Wild animals	No wild animals were used in the study.
Reporting on sex	Yes - we only used female mice.
Field-collected samples	No field collected samples were used in the study.
Ethics oversight	The care and use of all mice in this study were in accordance with the UK Animals in Science Regulation Unit's Code of Practice for the Housing and Care of Animals Bred, Supplied or Used for Scientific Purposes, the Animals (Scientific Procedures) Act 1986, and all procedures were performed under a UK Home Office Project license (PP8090463), which was reviewed and approved by the University of Cambridge Animal Welfare and Ethical Review Body.

Note that full information on the approval of the study protocol must also be provided in the manuscript.

Plants

Seed stocks	*Report on the source of all seed stocks or other plant material used. If applicable, state the seed stock centre and catalogue number. If plant specimens were collected from the field, describe the collection location, date and sampling procedures.*
Novel plant genotypes	*Describe the methods by which all novel plant genotypes were produced. This includes those generated by transgenic approaches, gene editing, chemical/radiation-based mutagenesis and hybridization. For transgenic lines, describe the transformation method, the number of independent lines analyzed and the generation upon which experiments were performed. For gene-edited lines, describe the editor used, the endogenous sequence targeted for editing, the targeting guide RNA sequence (if applicable) and how the editor was applied.*
Authentication	*Describe any authentication procedures for each seed stock used or novel genotype generated. Describe any experiments used to assess the effect of a mutation and, where applicable, how potential secondary effects (e.g. second site T-DNA insertions, mosiacism, off-target gene editing) were examined.*

Flow Cytometry

Plots

Confirm that:

☒ The axis labels state the marker and fluorochrome used (e.g. CD4-FITC).

☒ The axis scales are clearly visible. Include numbers along axes only for bottom left plot of group (a 'group' is an analysis of identical markers).

☒ All plots are contour plots with outliers or pseudocolor plots.

☒ A numerical value for number of cells or percentage (with statistics) is provided.

Methodology

Sample preparation	As above cell lines were used for analysis. These cells were simply dissociated in trypsin, resuspended in PBS and then subjected to FACS. GFP/BFP was excited with 488nm lazers.
Instrument	CytoFLEX flow cytometer (Beckman Coulter) and analysed with FCS Express v7.22.0031
Software	FCS Express v7.22.0031
Cell population abundance	Cell populations were always higher than 10% making for easy and accurate quantification.
Gating strategy	This is provided in Supplementary Fig 6. We used uninfected cells to establish our control gates. First, we selected cells based on their forward scatter and side scatter properties to exclude dead cells and debris. Next, we gated for singlets and excluded doublets by analysing the FSC-W vs. FSC-A plot. The fluorescence of BFP and GFP cell populations was detected following excitation with the 405 nm and 488 nm lasers, respectively.

☒ Tick this box to confirm that a figure exemplifying the gating strategy is provided in the Supplementary Information.
