# Supplementary material for: The synthetic lethal interaction between CDS1 and CDS2 is a vulnerability in uveal melanoma and across multiple tumor types
Source: Nat Genet. 2025 Jul 4;57(7):1672–83. doi: 10.1038/s41588-025-02222-1 (PMC12283370; doi:10.1038/s41588-025-02222-1)
Supplement: Supplementary file 8 — Uncropped blots. [file 41588_2025_2222_MOESM8_ESM.pdf]

Uncropped blots for extended data Figure 1

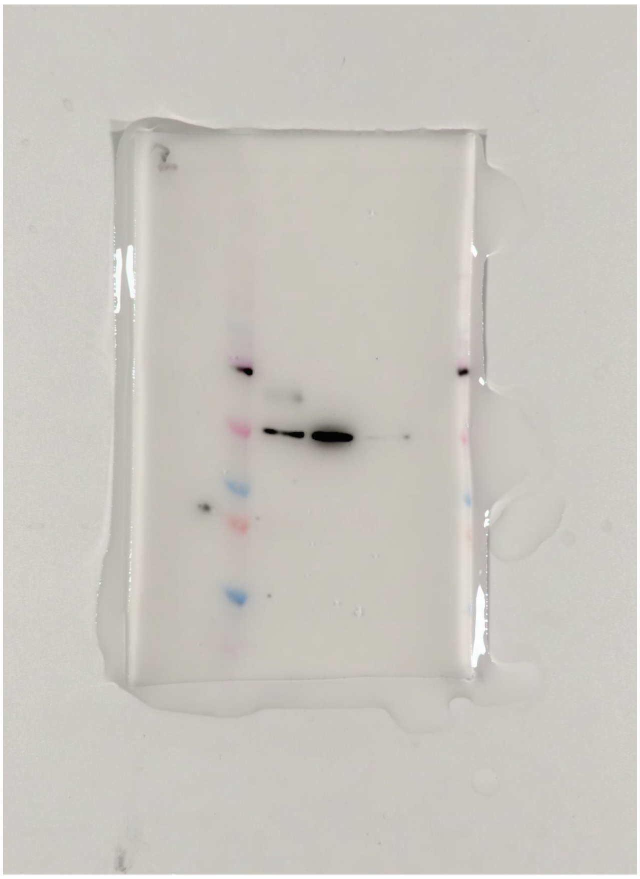

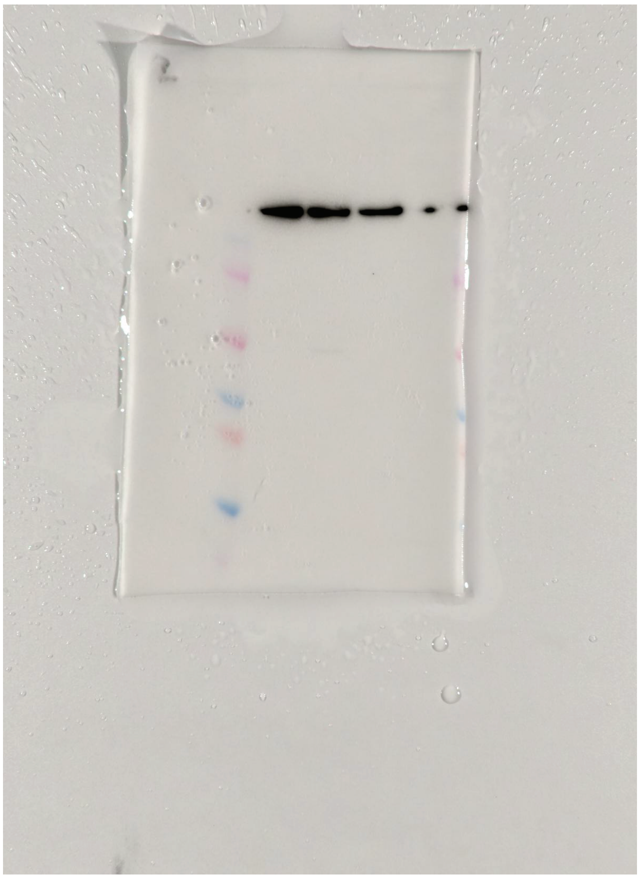

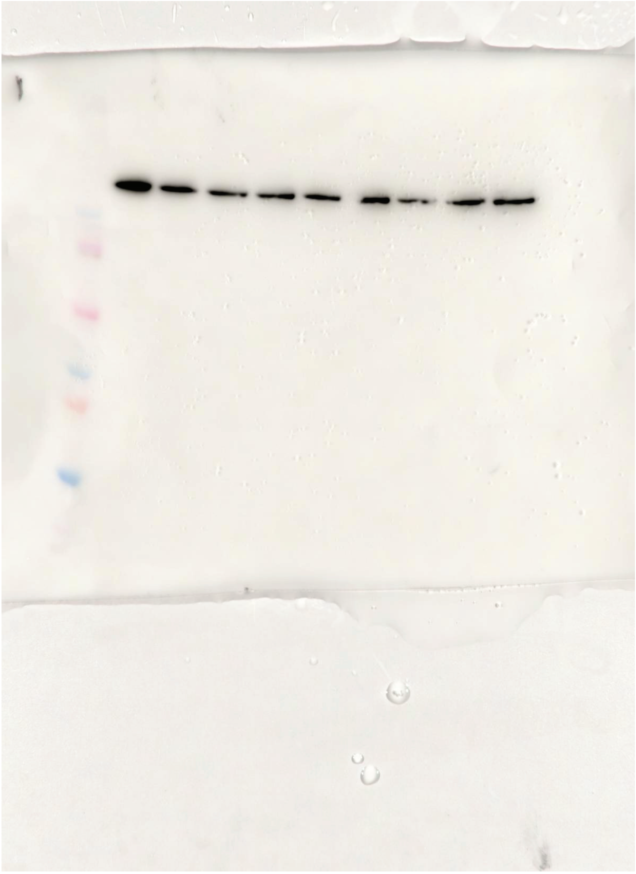

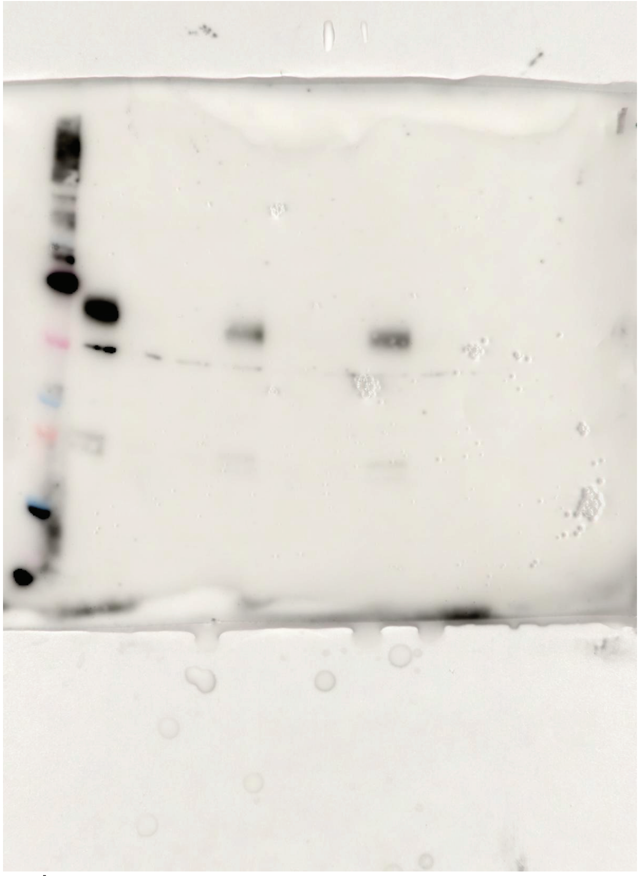

The Markers used are Thermo Novex Sharp from ThermoFisher
